# Electroconvulsive therapy in Germany: a nationwide multilevel analysis of determinants, regional patterns, and institutional variation

**DOI:** 10.1007/s00406-026-02305-y

**Published:** 2026-06-26

**Authors:** Marie Dreger, Andreas Uphaus, David Zilles-Wegner, Alexander Sartorius, Michael Guhra, Stefan Kreisel

**Affiliations:** 1 Department of Business and Health, Bielefeld University of Applied Sciences and Arts (HSBI), Bielefeld, Germany; 2 Medical School and University Medical Center OWL, Protestant Hospital of the Bethel Foundation, Department of Psychiatry and Psychotherapy, Bielefeld University, Bielefeld, Germany; 3 Department of Psychiatry and Psychotherapy, University Medical Center Göttingen, Göttingen, Germany; 4 Department of Psychiatry and Psychotherapy, Research Group Brain Stimulation, Central Institute of Mental Health, Medical Faculty Mannheim, Heidelberg University, Heidelberg, Germany; 5 DZPG, German Center for Mental Health, Partner Site Mannheim, Heidelberg, Ulm, Mannheim, Germany

**Keywords:** Electroconvulsive therapy, Administrative data, Multilevel analysis, Regional variation, Health services research, Germany

## Abstract

Electroconvulsive therapy (ECT) is an effective treatment for severe psychiatric disorders, yet its use may be shaped not only by clinical need but also by structural and institutional factors. Using nationwide 2023 administrative data from the German psychiatric prospective payment system (PEPP), we examined whether regional ECT utilization corresponded to regional diagnostic burden and assessed patient-, institutional-, and regional-level factors associated with receiving ECT. The analysis included 837,762 inpatient psychiatric discharges; in addition, descriptive information on day hospital ECT activity was obtained from publicly available aggregate data. Across inpatient and day hospital settings, 16,312 cases received ECT and 72,193 ECT procedures were documented. Regional ECT rates showed essentially no association with the regional frequency of ECT-relevant diagnoses. ECT provision was also strongly concentrated in larger institutions. In multilevel models, depression, catatonia, schizophrenia-spectrum disorders, bipolar disorder, female sex, older age, and annual psychiatric case volume were positively associated with ECT receipt. However, substantial residual heterogeneity remained at the institutional level (median odds ratio 23.6) after adjustment for case mix and contextual factors. These findings suggest that variation in ECT use in Germany is only weakly aligned with proxies of clinical need and is shaped to a considerable extent by institutional and structural conditions, indicating potential inequities in access to ECT across regions and providers.

**Supplementary Information:**

The online version contains supplementary material available at https://doi.org/10.1007/s00406-026-02305-y.

## Introduction

Electroconvulsive therapy (ECT) remains one of the most effective biological treatments in psychiatry for severe mental disorders. Its clinical role is particularly well established for severe depressive episodes and catatonia, and it continues to be considered an important treatment option when rapid symptom improvement is needed or when other interventions have not been sufficiently effective [1–3]. This position is reflected in national and international guidance documents [4–8]. Yet a robust evidence base does not necessarily translate into uniform access or implementation [9]. International work has repeatedly shown that ECT use varies considerably across countries, regions, and providers, raising the question of whether access to this evidence-based treatment is determined by patient need alone or also by structural and institutional features of mental health care delivery [10].

This question is highly relevant from both a clinical and a health services perspective. If the use of ECT closely tracks the distribution of severe and ECT-relevant psychiatric morbidity, geographic and institutional variation may largely reflect differences in clinical demand. If, however, marked variation persists after accounting for diagnostic burden and patient characteristics, disparities in delivery are more likely to indicate differences in service organization, provider readiness, local treatment cultures, or barriers to access, consistent with broader concepts of unwarranted variation in health care [11, 12]. ECT is therefore not only a therapeutic procedure, but also a useful lens through which broader issues of implementation, service provision, and equity in psychiatric care can be studied [10, 11].

A growing international literature based on routine data, national registries, and surveys suggests that such variation is substantial. Population-based analyses from Sweden documented nationwide utilization patterns of ECT and showed clear differences in patient profiles and treatment provision across settings [13]. Danish register-based studies similarly demonstrated temporal and regional variation in ECT use at the national level, underscoring that utilization is neither static nor evenly distributed even within a tax-funded healthcare system [14, 15]. In France, a nationwide study of psychiatric inpatients found that differences in ECT use were linked not only to case mix but also to health care supply, thereby highlighting the importance of contextual and provider-related determinants beyond clinical indication alone [11]. National reports and surveys from other countries also point to considerable heterogeneity in how ECT is organized and delivered, including Norway, Switzerland, Poland, Canada, and Australia [16–20].

Recent work from Germany has further suggested that the implementation of ECT remains uneven even where clinical need is considered substantial. Timäus et al. [21] reported growing demand for ECT, limited use in day-care settings, and frequent delivery of maintenance ECT in inpatient care, pointing to a service structure that remains closely tied to specialized institutional settings. More recently, Besse et al. [22] showed in forensic psychiatric hospitals in Germany and Switzerland that a substantial proportion of patients were considered to have an indication for ECT, whereas only a much smaller number actually received treatment, with lack of infrastructure identified as a major barrier. Together, these findings suggest that the real-world delivery of ECT may be constrained not only by indication, but also by organizational capacity and access pathways.

The reasons for such variation are likely multifactorial. At the service level, ECT requires specific clinical infrastructure, including trained psychiatrists, anesthesia support, recovery pathways, and institutional routines that enable repeated treatment sessions to be delivered safely and efficiently. These requirements make ECT particularly sensitive to local resource availability and organizational priorities, which may differ considerably across institutions and regions [11, 23, 24]. Survey-based evidence further suggests that the uptake and provision of neurostimulation treatments are shaped by perceived barriers among clinicians, patients, caregivers, and the general public, including concerns about safety, stigma, and knowledge gaps regarding indications and outcomes [9, 25–27]. Recent discussion in this journal has likewise pointed to broader gaps in familiarity with evidence-based psychiatric treatment options, including ECT, and to possible discrepancies between evidence, guideline recommendations, and professional practice [28]. Additional constraints may arise from the geographic concentration of ECT services. Travel distance and spatial availability are not trivial logistical details but potentially relevant determinants of who receives treatment and when [29, 30].

Against this background, the key unresolved question is whether observed variation in ECT use primarily reflects differences in clinical need or instead differences in access, service organization, and institutional practice. Administrative data from the German psychiatric prospective payment system (PEPP) provide a suitable basis to address these questions on a nationwide scale, as they cover inpatient psychiatric treatment across Germany**.** Using these data, the present study pursued two aims. First, we examined whether regional variation in ECT utilization corresponded to the regional frequency of diagnoses commonly associated with ECT use. Second, we analysed in a multilevel regression analysis which patient, institutional, and regional factors were associated with receiving ECT and investigated whether differences in its use depended more on the institution providing care or on the federal state in which patients were treated.

## Methods

### Study design and data sources

We conducted a nationwide cross-sectional study using 2023 administrative data from the German PEPP database accessed on-site through the Research Data Centre of the Federal Statistical Office and the statistical offices of the federal states [31]. The PEPP database captures routine reimbursement data for psychiatric and psychosomatic inpatient care in Germany and therefore provides a suitable basis for nationwide analyses of ECT utilization in routine practice. To complement the inpatient case-level analyses, we additionally used publicly available aggregate data from the Institute for the Hospital Remuneration System (InEK) for descriptive information on ECT activity outside the fully inpatient setting in Germany [32]. Population denominators for state-level rates were obtained from the Federal Statistical Office [33]. Regional contextual variables were derived from INKAR, the German system of spatial and urban development indicators maintained by the Federal Institute for Research on Building, Urban Affairs and Spatial Development [34].

### Study population and unit of analysis

The analytical population comprised all inpatient psychiatric discharges with a discharge date in 2023 recorded in the PEPP dataset [31]. The unit of analysis was the inpatient treatment episode rather than the individual patient. As a consequence, repeated admissions of the same person could not be linked across hospital stays. Aggregate InEK data on partial inpatient ECT were used descriptively only and were not included in the individual-level regression analyses [32].

### Outcome definition

The primary outcome was receipt of ECT during the inpatient stay. ECT was identified through the presence of at least one procedure code (Operationen- und Prozedurenschlüssel, OPS) from the 8–630.* code family in the discharge record [31]. For descriptive purposes, we also summarized documented ECT basic procedures and treatment sessions according to the corresponding OPS subcodes where the basic procedure is typically coded once per case, while treatment sessions reflect the number of individual ECT applications. The main regression analyses, however, used a binary indicator of any ECT versus no ECT during the inpatient episode.

### Diagnostic groups and covariates

We included patient-level, institutional-level, and regional covariates in order to examine whether ECT receipt was associated with case mix, provider characteristics, and contextual factors. Patient-level covariates comprised sex, age group, length of stay, and diagnosis categories relevant to ECT use. ECT-relevant diagnostic categories were derived from ICD-10 codes recorded in the administrative data and were defined on the basis of published evidence and clinical expertise [35, 36]. Because multiple diagnoses could be coded for a single discharge, categories were not mutually exclusive. The full ICD-10 code lists are provided in the Supplementary Material Table S1.

At the institutional level, we included annual psychiatric case volume as a proxy for institutional size and service throughput. At the regional level, we included population density and unemployment rate as contextual indicators of the local service environment. Population density was considered at the municipality level and unemployment at the county level, based on INKAR data [34]. These variables were selected to capture broad structural differences between more urban and more rural settings as well as socioeconomic context.

### Regional analysis of utilization and diagnostic burden

To examine whether regional variation in ECT use corresponded to regional diagnostic burden, we compared state level ECT utilization rates with state level rates of diagnoses commonly associated with ECT use. ECT utilization was expressed as the number of ECT-treated cases per 100,000 inhabitants using ECT case counts and state population denominators [31, 33]. Diagnostic burden was operationalized using the frequency of the predefined ECT-relevant diagnosis groups in the administrative data. We examined the relationship between regional diagnostic rates and ECT utilization using a scatterplot with a linear trend line and report the correlation coefficient and coefficient of determination.

### Multilevel regression analysis

To investigate factors associated with receiving ECT at the individual case level, we estimated a multilevel logistic regression model with patient-level covariates as fixed effects and random intercepts for institutions and federal states, reflecting the possibility that differences in ECT use are shaped not only by patient case mix, but also by institution-specific treatment practices and broader regional context. Formally, the model can be written as:

$$logit\left(P\left(EC{T}_{ijk}=1\right)\right)={\beta}_{0}+{\beta}_{1}{X}_{ijk}+{u}_{j}+{v}_{k},$$

where $EC{T}_{ijk}$ indicates whether inpatient case *i*, treated in institution *j* and located in federal state *k*, received at least one ECT procedure during the stay; ${X}_{ijk}$ represents the vector of observed covariates; and ${u}_{j} \sim N(0, \sigma ^{2})$ and ${v}_{k} \sim N(0, \sigma^{2})$ denote the institution and federal state specific random intercepts, respectively. Results are reported as adjusted odds ratios with 95% confidence intervals. Between-institution and between-state heterogeneity were quantified using the estimated random-effect variances and derived measures including the intraclass correlation coefficient (ICC) and the median odds ratio (MOR). Variance components and additional model fit statistics are reported in the Supplementary Material Table S2.

Fixed effects included sex, age group, length of stay, diagnostic categories, annual institutional case volume, population density, and unemployment rate. Continuous institutional level and regional level variables were z-standardized prior to model estimation to improve model convergence and facilitate comparison of effect estimates. For these variables, adjusted odds ratios therefore reflect the change in the odds of receiving ECT associated with a one-standard-deviation increase in the respective covariate. Results are reported as adjusted odds ratios with 95% confidence intervals.

Residual heterogeneity was quantified using the variance components of the random effects and derived measures including the ICC, the MOR, and marginal and conditional R^2^. Together, these measures describe the extent to which variation in ECT receipt was explained by observed covariates and the extent to which it remained attributable to clustering at the institutional and federal-state levels. The main model was estimated on complete cases.

In sensitivity analyses, we evaluated a range of alternative model specifications, including different operationalizations of ECT use, restrictions to institutions with higher case volumes, alternative categorizations of key covariates, and models specifying federal states as fixed rather than random effects. We also examined associations among candidate covariates using correlation measures, variance inflation factors (VIF), and Cramér’s V to assess potential multicollinearity and redundancy, and evaluated sparse categories and potential quasi-separation with regard to model stability and interpretability [37, 38].

## Results

Table 1 summarizes overall ECT utilization and the analytic samples used in the study. Among 837,762 psychiatric inpatient discharges treated in psychiatric institutions recorded in the 2023 PEPP dataset, 10,509 received at least one ECT procedure during the hospital stay, corresponding to an inpatient ECT rate of 1.25% among psychiatric inpatients**.** In addition to these inpatient episodes, publicly available aggregate data indicated 5,803 day hospital cases with ECT. Across both settings, 16,312 cases received ECT overall, corresponding to an overall ECT rate of 1.57%. A total of 72,193 ECT procedures were documented, including 61,444 inpatient and 10,749 partial inpatient procedures. Among inpatient ECT-treated cases, the mean number of documented procedures per case was 5.8 (SD 5.7), with a median of 3 procedures (IQR 1–10). Of all documented procedures, 5,338 were coded as ECT basic procedures and 56,106 as treatment sessions. Overall, ECT was billed by 190 of 520 psychiatric institutions, indicating that provision remained concentrated in a minority of institutions.

**Table 1:** Descriptive overview of ECT utilization and analytic samples in Germany, 2023

Overall psychiatric care activity	Inpatient psychiatric discharges	837,762
	Day hospital psychiatric cases	202,116
	Total psychiatric cases across both settings	1,039,878
ECT-treated cases	Inpatient discharges with ECT, n (%)	10,509 (1.25)
	Partial inpatient cases with ECT, n (%)	5,803 (2.87)
	Total ECT-treated cases across both settings, n (%)	16,312 (1.57)
ECT procedures	Total documented ECT procedures	72,193
	Inpatient ECT procedures	61,444
	Partial inpatient ECT procedures	10,749
	Procedures delivered outside the fully inpatient setting, %	14.9
	ECT basic procedures (OPS 8–630.2), n	5,338
	ECT treatment sessions (OPS 8–630.3), n	56,106
Service provision	Psychiatric institutions in PEPP data, n	520
	Institutions billing ECT, n (%)	190 (36.5)
Analytic sample	Complete-case sample for multilevel model, n	794,907

**Fig. 1:**
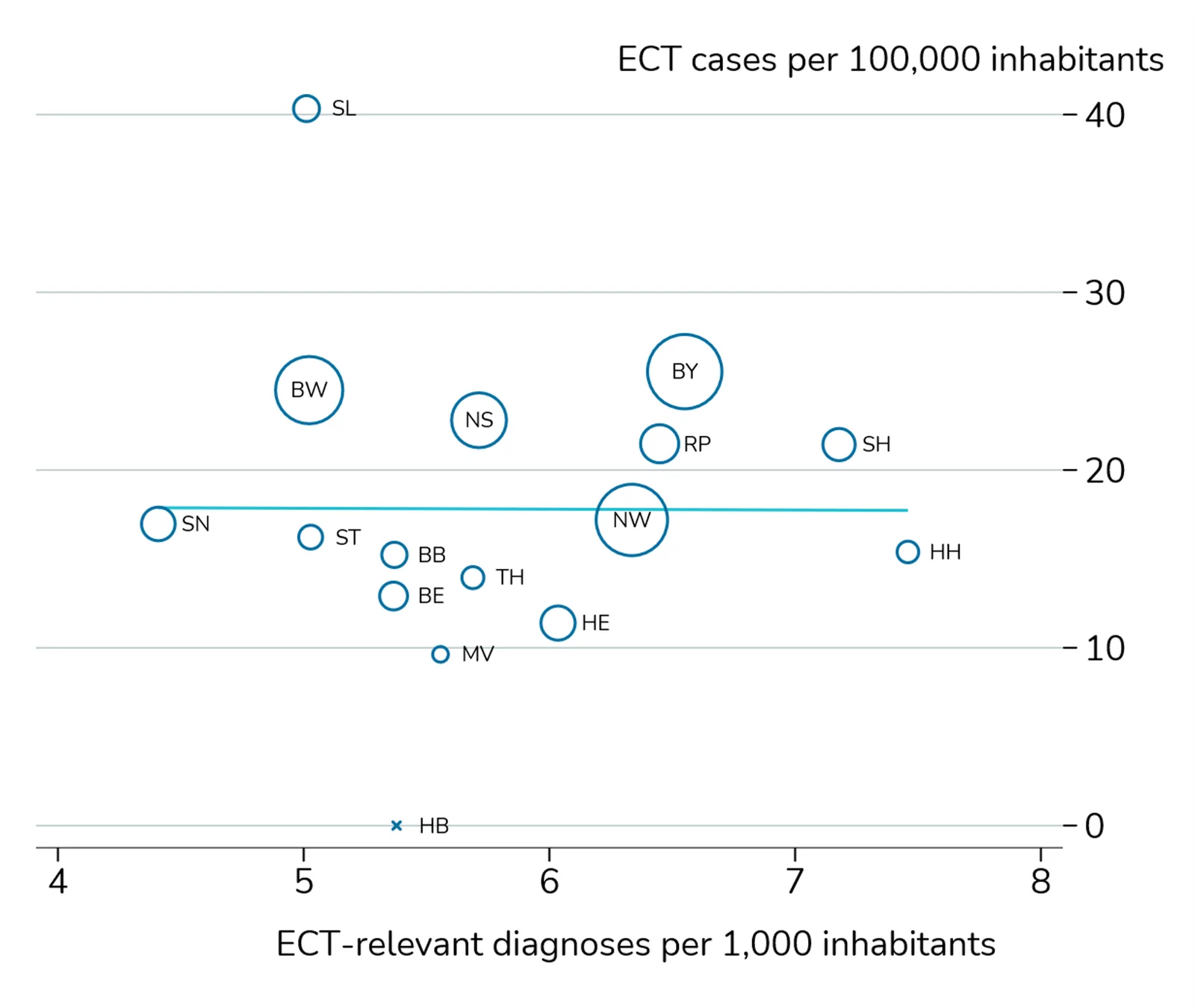
ECT rate in relation to the frequency of ECT-relevant diagnoses (defined in Supplementary Table S1). *BB*, Brandenburg; *BE*, Berlin; *BW*, Baden-Württemberg; *BY*, Bavaria; *HB*, Bremen; *HE*, Hessen; *HH*, Hamburg; *MV*, Mecklenburg-Western Pomerania; *NI*, Lower Saxony; *NW*, North Rhine-Westphalia; *RP*, Rhineland-Palatinate; *SH*, Schleswig–Holstein; *SL*, Saarland; *SN*, Saxony; *ST*, Saxony-Anhalt; *TH*, Thuringia. Bubble size corresponds to the absolute number of ECT cases per federal state; The regression line represents an unweighted linear fit across federal states

Within this descriptive context, the regional analyses showed that ECT use was not systematically aligned with regional diagnostic burden. Figure 1 compares the population-based rate of ECT-treated cases with the population-based rate of ECT-relevant diagnoses across federal states. The scatterplot revealed an almost flat regression line and virtually no linear association overall (r = − 0.0046, *R*^*2*^ ≈ 0). In other words, regions with a higher frequency of ECT-relevant diagnoses did not exhibit higher ECT utilization. The federal state Saarland stood out as a marked outlier with a particularly high ECT rate. However, excluding Saarland changed the overall interpretation only marginally. The association then became weakly positive (r = 0.236), but the explained variance remained very small (*R*^*2*^ ≈ 5.6%) and the relationship was not statistically meaningful. Thus, regional differences in ECT use cannot be understood as a reflection of regional morbidity.

**Fig. 2:**
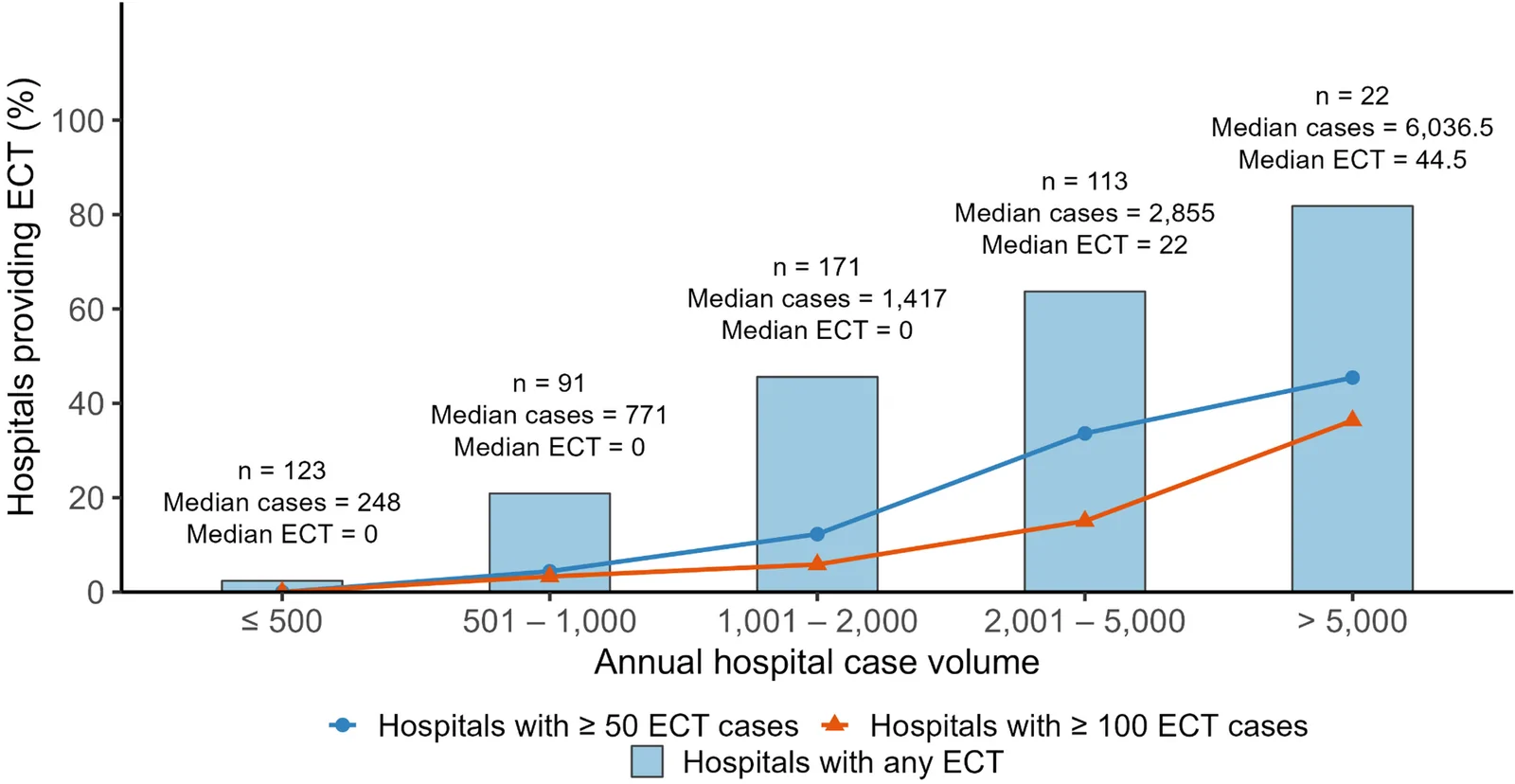
ECT provision across institution size categories in Germany, 2023. Bars show the share of hospitals providing any ECT; lines indicate higher ECT-volume thresholds

A further descriptive analysis showed that ECT provision was also strongly structured by institutional size (Fig. 2). The share of institutions providing any ECT increased markedly with annual psychiatric case volume, from 2.4% among institutions with 500 or fewer annual psychiatric cases to 81.8% among institutions with more than 5,000 annual psychiatric cases. Higher-volume ECT provision was likewise concentrated in larger institutions: institutions with at least 50 or 100 ECT cases were rare in smaller volume categories but became substantially more common among institutions with more than 2,000 annual psychiatric cases. Median ECT volume per hospital followed the same gradient, increasing from 0 in institutions with up to 2,000 annual psychiatric cases to 22 in institutions with 2001–5000 annual psychiatric cases and 44.5 in institutions with more than 5,000 annual psychiatric cases. Taken together, these patterns indicate that ECT provision in Germany was not only regionally uneven, but also structurally concentrated in larger institutions.

To examine whether these descriptive patterns were also reflected in adjusted associations at the individual case level, we next estimated a multilevel logistic regression model based on 794,907 inpatient discharges with complete information on all included covariates (Table 2). Diagnostic group was one of the strongest predictors of ECT receipt, with the highest odds observed for catatonia (aOR 12.32, 95% CI 10.36–14.66) and depression (aOR 10.01, 95% CI 9.35–10.73). Receiving ECT was also positively associated with schizophrenia-spectrum disorders (aOR 9.70, 95% CI 9.01–10.45) and bipolar disorder (aOR 2.07, 95% CI 1.91–2.25). These findings indicate that the likelihood of receiving ECT was markedly elevated in diagnostic groups that are clinically central to its use.

**Table 2:** Multilevel logistic regression analysis of factors associated with receiving ECT

Variable	Adjusted odds ratio (aOR)	95% confidence interval (CI)	p-value
*Sex* (reference: male)			
Female	1.25	1.19–1.31	< 0.001
*Age* (reference: 41–65 years)			
0–13	0.006	0.002–0.020	< 0.001
14–17	0.004	0.002–0.009	< 0.001
18–25	0.15	0.14–0.17	< 0.001
26–40	0.40	0.37–0.42	< 0.001
66–80	2.21	2.09–2.33	< 0.001
≥ 81	1.53	1.40–1.68	< 0.001
*Diagnosis* (reference: other)*			
Depression	10.01	9.35–10.73	< 0.001
Catatonia	12.32	10.36–14.66	< 0.001
Bipolar disorder	2.07	1.91–2.25	< 0.001
Schizophrenia-spectrum disorders	9.70	9.01–10.45	< 0.001
*Length of stay* (reference: 0–1 day)			
1–2 days	2.20	1.95–2.49	< 0.001
2–7 days	0.32	0.28–0.36	< 0.001
8–14 days	0.10	0.08–0.11	< 0.001
15–28 days	0.08	0.07–0.09	< 0.001
29–56 days	0.16	0.14–0.18	< 0.001
57–84 days	0.35	0.30–0.39	< 0.001
> 85 days	1.18	1.04–1.34	< 0.01
*Institutional characteristics*			
Annual psychiatric case volume (z-standardized)	3.67	2.09–6.44	< 0.001
*Regional characteristics* (municipality)			
Unemployment rate (z-standardized)	0.89	0.84–0.93	< 0.001
Population density (z-standardized)	0.81	0.77–0.85	< 0.001

*Diagnostic categories and corresponding ICD-10 codes are listed in Supplementary Table S1

Women had higher odds of receiving ECT than men (aOR 1.25, 95% CI 1.19–1.31). Relative to patients aged 41–65 years, the odds were substantially lower in younger age groups, including 0–13 years (aOR 0.006, 95% CI 0.002–0.020), 14–17 years (aOR 0.004, 95% CI 0.002–0.009), 18–25 years (aOR 0.15, 95% CI 0.14–0.17), and 26–40 years (aOR 0.40, 95% CI 0.37–0.42), whereas they were higher among patients aged 66–80 years (aOR 2.21, 95% CI 2.09–2.33) and still elevated among those aged 81 years or older (aOR 1.53, 95% CI 1.40–1.68).

Length of stay showed a distinctly non-linear pattern. Compared with very short admissions of 0–1 day, the odds of receiving ECT were highest for stays of 1–2 days (aOR 2.20, 95% CI 1.95–2.49), but markedly lower for intermediate stay categories, including 2–7 days (aOR 0.32, 95% CI 0.28–0.36), 8–14 days (aOR 0.10, 95% CI 0.08–0.11), 15–28 days (aOR 0.08, 95% CI 0.07–0.09), 29–56 days (aOR 0.16, 95% CI 0.14–0.18), and 57–84 days (aOR 0.35, 95% CI 0.30–0.39). Only stays longer than 85 days were again associated with slightly increased odds (aOR 1.18, 95% CI 1.04–1.34). At the institutional level, annual psychiatric case volume showed a strong positive association with ECT receipt (aOR 3.67 per standard deviation, 95% CI 2.09–6.44), consistent with the descriptive findings shown in Fig. 2, where both the likelihood of providing ECT and the occurrence of higher ECT volumes increased substantially with institutional psychiatric case volume. At the regional level, both unemployment rate (aOR 0.89, 95% CI 0.84–0.93) and population density (aOR 0.81, 95% CI 0.77–0.85) were inversely associated with receiving ECT.

Substantial unexplained heterogeneity remained after adjustment for all observed covariates (Supplementary Material Table S2). Random-effects analyses indicated that residual clustering was concentrated predominantly at the institutional rather than the federal-state level. The estimated variance was 10.97 (SD 3.31) for institutions and 0.60 (SD 0.78) for federal states, corresponding to an ICC of 0.74 and 0.04, respectively. The marginal R^2^ was 0.26, indicating that the fixed effects explained only a limited proportion of the total variation. After accounting for institution- and state-level clustering, the conditional R^2^ increased to 0.84, suggesting that a large share of overall variation was attributable to higher-level grouping, particularly at the institutional level. Together with the observed MOR of approximately 23.6, these findings indicate that otherwise similar patients could face markedly different probabilities of receiving ECT depending on the institution in which they were treated.

Findings were highly robust across sensitivity analyses, including alternative operationalizations of ECT use, restrictions to higher-volume institutions, alternative categorizations of key covariates, and models specifying federal states as fixed rather than random effects.

## Discussion

The central finding of this study is that variation in ECT use in Germany was not systematically aligned with regional diagnostic burden and therefore cannot be understood as a simple reflection of regional need. As shown in Fig. 1, the association between regional diagnostic rates and population based ECT utilization was essentially absent. The marked outlier position of Saarland warrants cautious interpretation, particularly because population-based rates in smaller federal states may be strongly influenced by the practice patterns of individual providers, local referral structures, or cross-regional catchment effects. Moreover, Saarland’s comparatively high utilization should not necessarily be interpreted as excessive use. As discussed below, international comparisons suggest that the regional pattern may also reflect comparatively low provision in several other German federal states, pointing to possible limited access or underuse. Importantly, excluding Saarland did not materially change the overall interpretation that ECT utilization was not systematically aligned with regional diagnostic burden. A brief nationwide report based on the same 2023 administrative dataset described the overall availability and utilization of ECT in Germany and showed that provision varied markedly across federal states and remained concentrated in a minority of psychiatric institutions [39]. The present analysis extends that descriptive account by examining whether regional ECT use corresponded to diagnostic burden and by quantifying the extent to which the remaining variation was structured at the institutional level.

The multilevel results suggest that clinically comparable patients may still face substantially different chances of receiving ECT depending on the institutions in which they are treated. The large institutional level variance, the high ICC at the institutional level, and the pronounced gap between marginal and conditional *R*^*2*^ all indicate that observed case mix and contextual covariates explained only part of the overall pattern. This interpretation is consistent with ECT-specific literature showing that treatment provision may be shaped not only by patient characteristics, but also by health care supply, infrastructure, and institutional implementation conditions [11, 22]. It is also compatible with survey-based evidence highlighting the relevance of staffing, clinical expertise, and local treatment culture for ECT provision [20, 23, 25].

A further descriptive finding reinforces this interpretation. ECT provision in Germany was strongly structured by institution size, with both the likelihood of offering ECT and the occurrence of higher ECT volumes increasing markedly across institution size categories (Fig. 2). This pattern may reflect structural and organisational barriers to ECT provision, particularly in smaller institutions, including the need for dedicated space, trained staff, and reliable anaesthesia support, which have been identified as obstacles to starting or expanding ECT services in provider surveys [40]. This descriptive concentration gradient is closely mirrored by the multivariable finding that annual psychiatric case volume was strongly positively associated with ECT receipt. Taken together, these results suggest that ECT provision in Germany is concentrated in a limited number of larger and presumably more experienced centers, rather than being broadly diffused across the inpatient sector. Compared with earlier German data, this pattern may also suggest that growth in ECT activity has been absorbed more by existing providers than by a broad expansion of provision across additional institutions [41].

At the same time, our findings indicate that ECT use is closely aligned with clinical indications. Accordingly, diagnosis was one of the strongest predictors of ECT receipt, with the highest adjusted odds observed for catatonia and depression, alongside markedly elevated odds for schizophrenia-spectrum disorders and bipolar disorder. These patterns are broadly consistent with guideline-based indications and suggest that, within treating institutions, ECT generally follows clinically plausible indication patterns. The female predominance observed in the present dataset [39] is in line with previous studies of ECT use [10, 13, 42, 43]. In the present multivariable analysis, however, the independent sex effect was only moderate after adjustment for diagnosis, age, and length of stay. This suggests that the descriptive overrepresentation of women is likely driven to a considerable extent by the distribution of clinical indications, particularly the higher prevalence of depressive disorders among women, rather than by a strongly sex-specific treatment practice in itself. Taken together, these findings suggest that while ECT use within institutions appears to be largely guided by clinical indications, substantial differences in access and implementation persist between institutions.

According to our data, Germany does not appear to be a country with either a particularly high or particularly low rate of ECT use when compared internationally. Based on all documented ECT-treated cases across settings, the present data correspond to approximately 19.5 ECT cases per 100,000 inhabitants, whereas the rate based on inpatient cases alone is about 12.6 per 100,000 inhabitants [32, 39]. These estimates are higher than earlier questionnaire-based figures reported for Germany and above rates described for Poland, Switzerland, France, and England, but they remain below those reported for Sweden, Denmark, Norway, and Australia [13, 14, 16, 17, 19, 20, 41, 42, 44, 45]. However, these comparisons should be interpreted cautiously. First, the present analysis was based on cases rather than individual patients, so population-based case rates may overestimate person-based treatment rates. Second, definitions, reporting periods, treatment settings, and denominators differ across countries.

A further finding concerns the treatment setting. Approximately 15% of documented ECT procedures were delivered outside the fully inpatient setting [32, 39]. This underlines the relevance of partial inpatient and ambulatory care pathways, which have so far received only limited attention in both routine ECT provision and health services research in Germany. At present, ECT in Germany still appears to be largely anchored in hospital-based structures, with limited implementation in ambulatory care. Internationally, the balance between treatment settings appears to be configured differently. In Switzerland, Sweden, and Canada, outpatient ECT has been described as the predominant mode of delivery, reaching proportions of up to 90% of all ECT applications in Canada [13, 20, 46]. Differences in treatment setting are therefore likely to reflect not only clinical custom, but also broader organizational and incentive structures within healthcare systems [47, 48].

At the same time, variation in ECT use that is not fully explained by diagnostic case-mix is unlikely to reflect structural factors alone. Although ECT is supported by a substantial evidence base and longstanding guideline recommendations, public and professional controversy persists and may still influence its implementation in routine care [1–3, 8, 9]. Patient-side factors may further contribute to this mismatch, as willingness to undergo ECT is shaped not only by clinical severity, but also by treatment-related expectations, concerns, and decision-making processes [27]. This underlines the importance of transparent, evidence-based information and shared decision-making to ensure that ECT is considered on the basis of clinical indication rather than being constrained by structural barriers, stigma, or misinformation.

Several limitations should be considered. First, the analysis was based on inpatient discharges rather than individual patients, so repeated admissions of the same person could not be linked across episodes. As a result, case-based population rates may overestimate person-based treatment rates. Second, administrative data do not capture several clinically important determinants of ECT indication and access, including symptom severity, treatment resistance, patient preference, referral pathways, and clinician attitudes. Third, although the multilevel analysis accounted for institutional-level clustering, the descriptive regional comparison was limited to federal state level and therefore could not fully represent finer-grained catchment structures or travel-time-based service access. At the same time, the study has important strengths. To our knowledge, this is the first nationwide analysis of ECT use in Germany based on administrative data that combines descriptive, spatial, and multilevel analytical approaches within a single framework.

Overall, the present findings suggest that the central issue in Germany is not simply how often ECT is used, but how unevenly its use is distributed across institutions and regions relative to likely need. The combination of weak alignment between diagnostic burden and ECT utilization, strong provider level clustering, and concentration of provision in a limited number of institutions suggests that organization, institutional routines, and locally embedded decision cultures play a major role in shaping access to ECT in Germany. For future research and service planning, the key issue is therefore how evenly and reliably access to it can be ensured across institutional settings.

## Supplementary Information

Below is the link to the electronic supplementary material.

Supplementary Material 1

## Data Availability

The administrative health data underlying this study cannot be shared publicly because they are subject to strict data protection and access restrictions. The analyses were conducted using on-site access to the Research Data Centre of the Federal Statistical Office and the statistical offices of the federal states (FDZ). Access for qualified researchers may be granted upon application to the FDZ and in accordance with applicable legal provisions. Additional aggregate data used in this study are publicly available from InEK and the Federal Statistical Office.
