# Supplementary material for: Electroconvulsive therapy in Germany: a nationwide multilevel analysis of determinants, regional patterns, and institutional variation
Source: Eur Arch Psychiatry Clin Neurosci. 2026 Jun 26;276(7):3459–68. doi: 10.1007/s00406-026-02305-y (PMC13562103; doi:10.1007/s00406-026-02305-y)
Supplement: Supplementary file 1 [file 406_2026_2305_MOESM1_ESM.docx]

**Title: Electroconvulsive therapy in Germany: a nationwide multilevel analysis of determinants,** **regional patterns, and institutional variation**

**Journal: European Archives of Psychiatry and Clinical Neuroscience**

Author information:

Marie Dreger^1,2^, Andreas Uphaus^1^, David Zilles-Wegner^3^, Alexander Sartorius^4,5^, Michael Guhra^2^, Stefan Kreisel^2^

^1^ Bielefeld University of Applied Sciences and Arts (HSBI), Department of Business and Health, Bielefeld, Germany

^2^ Department of Psychiatry and Psychotherapy, University Medical Center OWL, Evangelisches Klinikum Bethel, Bielefeld University, Bielefeld, Germany

^3^ Department of Psychiatry and Psychotherapy, University Medical Center Göttingen, Göttingen, Germany

^4^ Department of Psychiatry and Psychotherapy, Research Group Brain Stimulation, Central Institute of Mental Health, Medical Faculty Mannheim, Heidelberg University

^5^ DZPG, German Center for Mental Health, Partner site Mannheim/Heidelberg/Ulm (ZIHUb), Germany.

Corresponding author: Marie Dreger, [marie.dreger@hsbi.de](mailto:marie.dreger@hsbi.de)

Supplementary Table S1

**Tab. S1** Definition of diagnostic groups and corresponding ICD-10-GM codes

| **Diagnostic group*** | **ICD-10-GM codes** | **Source** |
| --- | --- | --- |
| Depression | F32.0–F32.3, F32.8–F32.9, F33.0–F33.3, F33.8–F33.9, F34.1, F41.2, F34.8–F34.9, F38.0–F38.1, F38.8, F39, F99, F20.4 | based on Fiest et al. 2014; extended |
| Catatonia | F06.1, F20.2 | authors’ definition |
| Bipolar disorder | F31.3–F31.6, F31.8–F31.9, F31.0–F31.2 | authors’ definition |
| Schizophrenia-spectrum disorders | F20.0–F20.3, F20.6, F20.8–F20.9, F25.0–F25.2, F25.8–F25.9 | based on Vernal et al. 2018; extended |

***Note**: Diagnostic groups were constructed based on ICD-10-GM codes. Categories may overlap because multiple diagnoses can be coded within a single discharge case.

Supplementary Table S2

**Tab. S2** Random effects and model fit

| **Metric** | **Value** |
| --- | --- |
| Hospital-level variance (IK) | 10.97 |
| Hospital-level standard deviation | 3.31 |
| Federal-state-level variance | 0.60 |
| Federal-state-level standard deviation | 0.78 |
| Hospital-level ICC | 0.74 |
| Federal-state-level ICC | 0.04 |
| Marginal R² | 0.26 |
| Conditional R² | 0.84 |
| Observations | 794,907 |
